# Production of short-chain fatty acids (SCFAs) as chemicals or substrates for microbes to obtain biochemicals

**DOI:** 10.1186/s13068-023-02349-5

**Published:** 2023-06-03

**Authors:** Elia Tomás-Pejó, Cristina González-Fernández, Silvia Greses, Christian Kennes, Nuria Otero-Logilde, María C. Veiga, David Bolzonella, Bettina Müller, Volkmar Passoth

**Affiliations:** 1 Biotechnological Processes Unit, IMDEA Energy, 28935 Móstoles, Madrid, Spain; 2 Department of Chemical Engineering and Environmental Technology, School of Industrial Engineering, University of Valladolid, Valladolid, Spain; 3 Institute of Sustainable Processes, Valladolid, Spain; 4 Chemical Engineering Laboratory, Faculty of Sciences and Center for Advanced Scientific Research, Centro de Investigaciones Científicas Avanzadas (CICA), BIOENGIN Group, University of La Coruña, E-15008 La Coruña, Spain; 5 Department of Biotechnology, University of Verona, Verona, Italy; 6 Department of Molecular Sciences, Swedish University of Agricultural Sciences, Box 7070, 75007 Uppsala, Sweden

**Keywords:** Carboxylic acids, Short-chain fatty acids, Biosynthesis, Anaerobic fermentation, Organic waste streams, Platform chemicals, Bioproducts, Microbial oils, Polyhydroxyalkanoates, Microbial electrolytic cells

## Abstract

Carboxylic acids have become interesting platform molecules in the last years due to their versatility to act as carbon sources for different microorganisms or as precursors for the chemical industry. Among carboxylic acids, short-chain fatty acids (SCFAs) such as acetic, propionic, butyric, valeric, and caproic acids can be biotechnologically produced in an anaerobic fermentation process from lignocellulose or other organic wastes of agricultural, industrial, or municipal origin. The biosynthesis of SCFAs is advantageous compared to chemical synthesis, since the latter relies on fossil-derived raw materials, expensive and toxic catalysts and harsh process conditions. This review article gives an overview on biosynthesis of SCFAs from complex waste products. Different applications of SCFAs are explored and how these acids can be considered as a source of bioproducts, aiming at the development of a circular economy. The use of SCFAs as platform molecules requires adequate concentration and separation processes that are also addressed in this review. Various microorganisms such as bacteria or oleaginous yeasts can efficiently use SCFA mixtures derived from anaerobic fermentation, an attribute that can be exploited in microbial electrolytic cells or to produce biopolymers such as microbial oils or polyhydroxyalkanoates. Promising technologies for the microbial conversion of SCFAs into bioproducts are outlined with recent examples, highlighting SCFAs as interesting platform molecules for the development of future bioeconomy.

## Background

Fossil raw materials are still the basis for producing fuels, chemicals, food, and feed for modern society. The dependency on increasingly scarce, non-renewable resources leads to declining security of supply and is a major reason for the release of greenhouse gases (GHG) and, thus for climate change.

Biomass is a renewable resource that has the potential to replace a significant portion of fossil resources [1]. Nevertheless, this replacement can result in competition for arable land, land-use changes of natural ecosystems, and in some cases, even increased GHG release compared to fossil-based systems [2]. One opportunity to overcome these difficulties at least partially can be the use of by-products, co-products, and organic waste from agriculture and forestry, i.e., non-edible lignocellulosic residues [2, 3], for the production of bioproducts.

Lignocellulose is the most abundant biomass on Earth, with an estimated annual production of about 181.5 billion tons. Lignocellulose consists mainly of the polymers cellulose and hemicellulose, which are polysaccharides, and lignin [4]. Although an abundant and, in most cases, cheap material, its conversion into biofuels, food, feed, and biochemicals is a costly process. This is mainly due to the resilience of the material, which requires energy-intensive pretreatment to release monosaccharides and other low molecular weight compounds for subsequent fermentative or chemical conversions [4–6]. The currently established pretreatment methods are mainly aimed at favouring the release of sugars, the most important monosaccharides being glucose from cellulose and, to a lesser extent, from hemicellulose, and xylose from hemicellulose.

Current biotechnologies implemented to generate bioproducts such as ethanol, microbial oils or other valuable compounds are based on the controlled cultivation of monocultures such as *Saccharomyces cerevisiae* or oleaginous yeasts, utilizing traditional sugar-based substrates. The bioprocess yields when using carbon sources such as xylose or glucose are well known; however, their use is associated with high costs and negative environmental impact [7].

The production of carboxylic acids instead of sugars from biomass may be an alternative or at least complementary approach to valorise waste materials. Carboxylic acids are organic acids that contain a carboxyl group (-COOH). Among them, especially short-chain fatty acids (SCFAs) are increasingly in the focus of biotechnological research, as they have a wide range of applications and can be generated from a variety of raw materials. SCFAs are fatty acids composed of six or fewer carbon atoms that can be distilled at atmospheric pressure [8].

SCFAs can be formed by a variety of different processes, such as during the thermochemical acid pretreatment of lignocellulosic biomass, which liberates acetyl residues from hemicellulose and lignin to form acetic acid [9]. However, the main bioprocess for the generation of SCFAs is based on the anaerobic fermentation (AF) of biomass. Here biopolymers are hydrolysed and the soluble monomers are mainly fermented to the SCFAs acetic, propionic, and butyric acid as well as to lactate, alcohols, and hydrogen. This is called acidogenic, AF, or dark fermentation [8].

Apart from lignocellulosic biomass, SCFAs can be produced via AF from a variety of different substrates, including sludge, food waste, the organic fraction of municipal solid waste, and paper mill effluents. By modifying the fermentation condition in terms of temperature, pH, hydraulic and solid retention time (HRT and SRT), organic loading rate (OLR), and feedstock composition, it is possible to modify product yield, production rate, and the SCFAs profile obtained [8, 10].

Due to their functional group, SCFAs are extremely useful for the chemical industry since carboxylic acids are precursors of reduced chemicals and derivatives (esters, ketones, aldehydes, alcohols, and alkanes) in conventional organic chemistry. In addition, they are also well-known substrates for the production of biofuels, such as methane and hydrogen, as well as other biopolymers, such as microbial oils or polyhydroxyalkanoates (PHAs) [11].

This review provides a survey about the production of SCFAs from low-value waste products and their potential biotechnological applications. Conditions favouring SCFAs production via biotechnological routes and their advantages over chemical synthesis have been identified. In addition, different approaches using SCFAs as carbon sources for different microorganisms are critically discussed.

## Chemical synthesis of SCFAs

Acetic acid (C2) is one of the most important commodity chemicals for the chemical and food industries. In 2020, the global acetic acid market was 6.9 billion USD and it is expected to grow above USD 15 billion by 2027. Its market price is rather variable, presently between about €1300 (i.e., USD 1300) and €1800 (i.e., USD 1800) per ton, depending on fluctuating oil prices or, also, pandemic situations. The majority of commercial acetic acid is produced from petroleum-derived compounds by chemical synthesis, with carbonylation of methanol being one of the most common petroleum-based processes for producing acetic acid [12, 13]. The catalytic carbonylation of methanol to acetic acid corresponds to the following overall reaction (14):

$${\text{CH}}_{{3}} {\text{OH}} + {\text{CO}} \to {\text{CH}}_{{3}} {\text{COOH}}$$

The CO required for this reaction can be obtained from synthesis gas (i.e., CO and H_2_, mainly), as in many other catalytic production processes, e.g., the production of various alcohols [15, 16]. The methanol used in the catalytic production of acetic acid is also often obtained from syngas. Recent research addressed the feasibility of using the greenhouse gas CO_2_ (and H_2_), instead of CO, for the catalytic conversion of methanol, according to the following reaction:

$${\text{CH}}_{{3}} {\text{OH}} + {\text{CO}}_{{2}} + {\text{ H}}_{{2}} \to {\text{CH}}_{{3}} {\text{COOH }} + {\text{ H}}_{{2}} {\text{O}}$$

However, this process is still in its infancy and needs substantial further research and improvements. In most cases, homogenous rhodium or iridium catalysts are used, but research is ongoing to find improved catalysts with higher catalytic activities and better stability and to optimize the operating conditions. The conventional chemical process for acetic acid production also requires a halogen promoter, typically methyl iodide. Drawbacks of this chemical process are that rhodium catalysts are very expensive and iodine is highly corrosive. Heterogenous catalytic processes are also being studied, but still need further improvements [14].

Syngas can actually be used both for the thermochemical synthesis of acetic acid [17] as well as for its bioproduction, which has been reviewed and compared recently in more detail elsewhere [18], and a detailed overview is beyond the scope of this manuscript. Thus, similar feedstocks are suitable in both cases when those processes are based on syngas conversion. The biological syngas process is catalysed by specific anaerobic bacteria converting one-carbon (C1) gases into acetic acid or longer chain organic acids such as butyric or caproic acids [19]. Those acids can eventually even further be converted to other biofuels or biochemicals (e.g., bioalcohols) if desired.

Propionic acid (C3) is used by the plastic, pharmaceutical, and cosmetics industries. In 2020, the global propionic acid market was valued at 1.1 billion USD and is expected to reach 1.4 billion USD by 2028. (https://www.verifiedmarketresearch.com/product/propionic-acid-market/, accessed 2022–08-29). Propionic acid is industrially synthesized by petrochemical processes, which converts mainly ethylene, carbon monoxide and steam (Reppe process) or ethanol and carbon monoxide (Larson process) in the presence of boron trifluoride into propionic acid [20].

Butyric acid (C4) has many applications in pharmaceutical, food, cosmetics and chemical industry. The butyric acid derivatives market was valued at 450.9 million USD in 2020 and is expected to grow by 6.7% until 2028 (https://www.verifiedmarketresearch.com/product/butyric-acid-derivatives-market/, accessed 2022–08-29). Butyric acid can be produced through petroleum-based catalytic processes, but as for most acids produced through catalytic processes, its synthesis also requires higher temperatures and pressures than room temperature and -pressure. One common chemical route to obtain butyric acid is through the chemical oxidation of butyraldehyde (i.e., butanal). The latter can be obtained from crude oil through oxosynthesis with propylene as starting reagent [18]. This is known as the oxoreaction, involving propylene and syngas.

Valeric acid (C5) is produced from butylene and syngas and is used in the food and cosmetics industries [19]. Industrial synthesis processes for the production of caproic acid (C6) use 1-hexanol or cyclohexanol from petroleum processing as starting materials, which are converted to caproic acid by means of oxidative processes (https://www.internetchemie.info/chemie-lexikon/stoffe/c/capronsaeure.php).

Caproic acid is used in the pharmaceutical, food and cosmetics industries. The market is projected to grow 5.6% by 2027, to 253 million USD (https://www.researchandmarkets.com/reports/5302114/caproic-acid-global-market-trajectory, accessed 2022–08-29).

## Biosynthesis of SCFAs

**Fig. 1:**
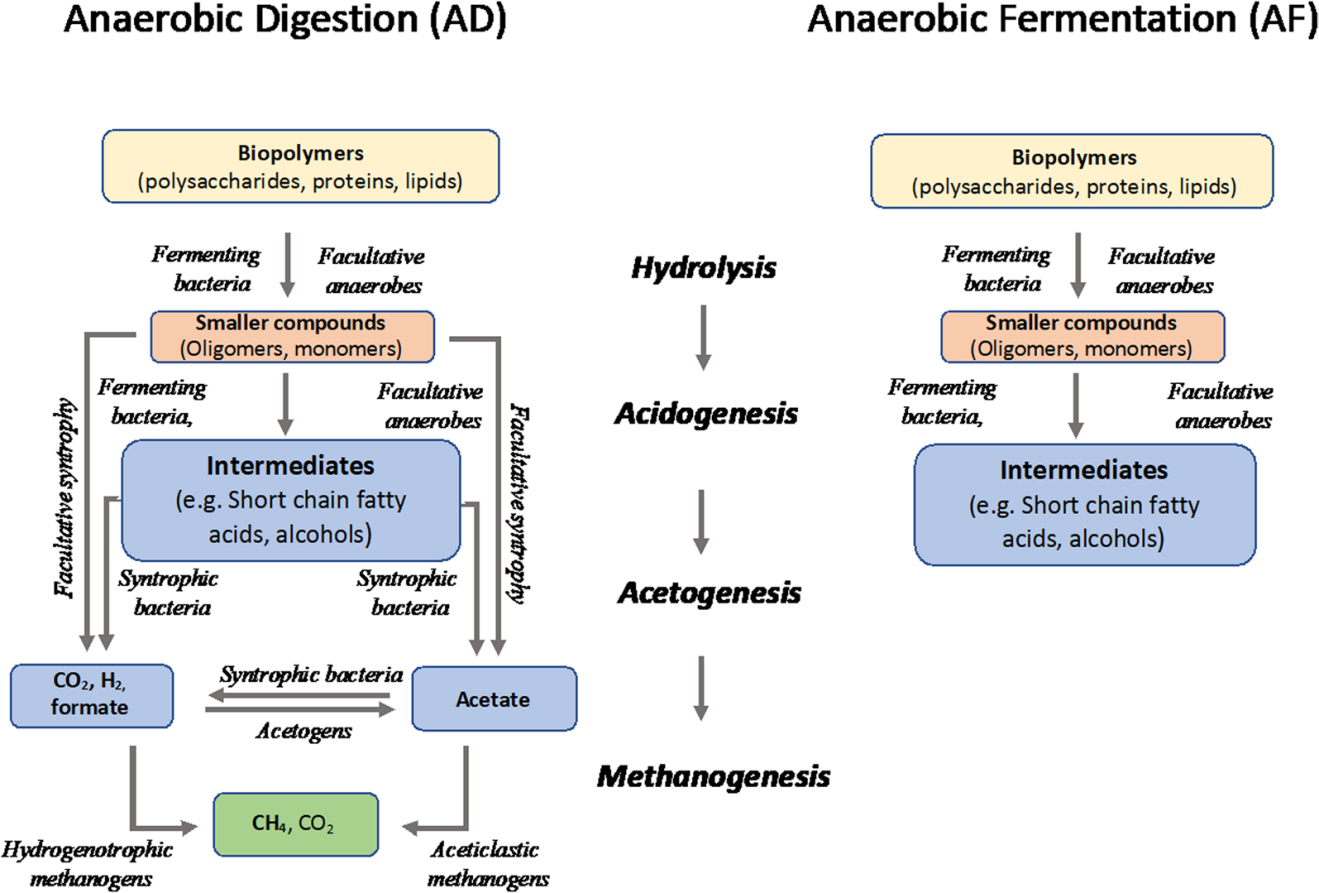
General scheme of steps involved in both AD (with biogas as the final product) and AF (with mainly SCFAs as final products). Complex organic matter is degraded by hydrolytic enzymes produced by fermenting and facultative anaerobic bacteria (Hydrolysis). Hydrolysed products are fermented to volatile SCFAs such as acetic, propionic, butyric, valeric and caproic acids as well as alcohols, longer chain fatty acids, lactate, CO_2_, and formate (Acidogenesis). The hydrolysing and fermenting bacteria belong to the phyla Firmicutes, Bacteriodetes, Spirochaetes, Actinobacteria, Chloroflexi, and Proteobacteria. Intermediates are further metabolized to methanogenic substrates H_2_, formate, and acetate due to the activity of specialized syntrophic consortia as well as acetogens (acetogenesis). Hydrogenotrophic and acetoclastic methanogens complete the process by converting these compounds to methane and CO_2_ (methanogenesis) [21]. In order to accumulate SCFAs acetogenesis and methanogenesis must be prevented. SCFAs = short-chain fatty acids

As introduced, SCFAs are intermediate compounds synthesized during anaerobic digestion (AD) of organic substrates. This anaerobic food chain requires the harmonized activities of a large number of physiological diverse microorganisms and leads finally to the production of methane and carbon dioxide [21]. Initially, the polymeric substrates such as polysaccharides, proteins, and lipids are hydrolysed (hydrolysis). The hydrolysed products are fermented to SCFAs, longer-chain fatty acids, lactate, alcohols, CO_2_, formate, and H_2_ (acidogenesis). These intermediates are then metabolized to the methanogenic substrates: acetate, H_2_, and formate (acetogenesis), which are further converted to methane and carbon dioxide (methanogenesis) (Fig. 1). Both substrate composition and process conditions determine the microbial composition and community structure ultimately affecting intermediary SCFAs concentration and profile [20].

In order to accumulate SCFAs, the last two steps of anaerobic digestion, acetogenesis and methanogenesis, must be prevented, resulting in a shortened process known as AF (Fig. 1).

SCFAs derived from organic wastes via AF can serve as low-cost carbon sources for a variety of microorganisms to produce, e.g., microbial oils or other high-value compounds. This innovative approach has recently attracted high interest since it allows transforming a low-value residue into an added-value product, which increases the cost-effectiveness of the process and GHG savings. Nevertheless, valorizing complex organic waste via AF results in SCFA production with varying acid profiles (C2 to C6) which definitely affects the microbial downstream processes [22]: For instance, high acetic acid (C2) concentration in the SCFA pool promotes yeast growth and lipids accumulation. However, an opposite trend has been observed when caproic acid (C6) dominates the SCFAs profile. Different authors suggested that it may act as a bioprocess inhibitor [23]. Given the high importance of the SCFA profile for the metabolic performance of microorganisms, different strategies have been developed to control the specific SCFA production based on mainly two process factors: the macromolecular composition of the organic waste and the operational conditions imposed in AF.

## Bioreactor conditions favouring AF and SCFA production

As previously mentioned, the specific SCFAs profile is highly dependent on the feedstock composition, with the operational conditions in the bioreactor affecting the microbial composition and structure. The operational conditions used in AF bioreactors thus play a key role in process efficiency as they influence the development of the microbial community and thus determine the dominance of metabolic pathways and their end products. For example, the use of high organic loading rates (OLR) and short hydraulic residence times (HRT) promotes the growth of hydrolytic and acidogenic bacteria, while the slow-growing methanogens are washed out, thus greatly reducing further SCFAs degradation. Likewise, the process pH and temperature affect the physiology and activities of the various metabolic groups differently. In this regard, AF conditions have to be properly selected. The following sections briefly summarize which AF conditions promote hydrolytic and acidogenic activity, reducing acetogenic and methanogenic activities, and how this affects the SCFAs profile.

### Process pH

Conventionally, methanogenic activity requires a neutral pH, whereas acidogenesis is enhanced at pH values between 5.5 and 6.0. Slightly acidic pH has been successfully applied in AF of carbohydrate-rich residues to limit methanogenic activity and increase SCFAs accumulation, reaching high acidification yields (60–80% of the soluble chemical oxygen demand) and bioconversion efficiency (30–60%) [24–26]. In this regard, the natural acidification of food waste or agricultural residues can be beneficial to lower the process pH. However, pH values lower than 5.5 must be avoided as both methanogenic and acidogenic activities are inhibited, favouring the establishment of metabolic pathways that accumulate primary metabolites such as lactic acid or ethanol [27].

On contrary, alkaline pH promotes the degradation of protein-rich feedstock. For instance, Bermúdez-Penabad et al. (2017) [28] demonstrated that pH 8 favours the bioconversion of tuna waste into SCFAs and identified a very high alkaline pH [10–12] as the limiting condition. Such high pH values notably enhanced the hydrolysis of the organic matter but negatively affected the acidogenic activity, resulting in a lower acidification yield.

The process pH affects not only the feedstock degradability, but also the SCFAs profile. In this sense, butyric acid concentration normally increases at alkaline pH, while acetic and propionic acids prevail when the AF is performed at acid pH [5, 6, 26].

### Hydraulic retention time and organic loading rate

Hydraulic retention time (HRT) and organic loading rate (OLR) are two operational parameters that are usually interconnected. HRT adjustment not only controls the growth of microorganisms but also the amount of organic matter loaded into the reactor. Extended HRT promotes the degradation of complex feedstock as it allows higher bacterial growth and the establishment of a microbial community capable of hydrolysing the components of the feedstock. This in turn promotes the availability of soluble organic matter for the acidogenic step. However, a long HRT involves low flow rates and thus promotes microorganisms with low growth rates, such as the non-desired methanogenic archaea. Therefore, short HRT is preferred to reduce methanogenic activity. The use of short HRTs is also related to a high OLR, which generates a process overload that reduces the process pH and thus promotes SCFAs production and reduces methanogenic activity as described above. Cavinato et al. (2017) [24] found that HRT shorter than 6 days boosted SCFAs production, while Teixeira et al. (2020) [29] found that increasing HRT from 19 to 41 days resulted in a two-fold increase in SCFAs production when using a complex substrate as feedstock without pre-treatment. In addition, short HRT is usually associated with the production of the shorter SCFAs, acetic and propionic acids [26], while long HRT of carbohydrate-rich feedstock combined with acidic pH leads to accumulation of the longer SCFAs, butyric and caproic acids [30]. Therefore, choosing the right operational conditions depends on the macromolecular composition of the feedstock and the SCFAs profile desired for the subsequent processes.

Given the high influence of HRT and OLR on process performance and SCFAs profile, alternative reactor configurations to continuous stirred tank reactors (CSTR) are under intense investigation. Based on feedstock features, sequencing batch reactor (SBR), upflow anaerobic sludge blanket (UASB), and anaerobic membrane bioreactor (AnMBR) are being studied since these configurations allow decoupling the HRT and the solid retention time (SRT). Thus, a high flow rate can be fed into the reactor without significantly enlarging the tank volume due to the high solid retention in the system, increasing the cost-effectiveness of the process.

### Process temperature

The process temperature has a significant impact on the metabolic rates of microorganisms. As with pH, the optimal temperature range is determined by the microbiota selected on the basis of the composition and complexity of the feedstock. Since hydrolysis is usually the limiting step of the AF process, mesophilic and thermophilic processes are commonly used to promote microbial enzymatic activities. Mesophilic processes run at temperatures from 20 to 43 °C, with 35–37 °C usually considered optimal. Thermophilic processes are performed at 50–60 °C [31]. Mesophilic processes have been found to be optimal for feedstocks such as cow manure, maize silage, or the organic fraction of municipal solid waste, as both hydrolytic and acidogenic activities are boosted [24, 32]. On the other hand, thermophilic processes are commonly used in AF of recalcitrant feedstock such as protein-rich substrates [33]. Although thermophilic processes involve high energy demand, high bioconversion efficiencies can be achieved with a highly specific SCFAs profile, simplifying downstream processes [34].

Basically, both mesophilic and thermophilic operational conditions promote the growth of methanogenic archaea, which provokes a conversion of SCFAs towards methane production and thus reduces the AF efficiency. To overcome this situation, high process temperatures must be carefully combined with more restrictive operational parameters such as short hydraulic retention times and acidic pH.

Feedstocks, on the other hand, which are characterized by a high content of readily biodegradable carbohydrates, can also be successfully converted into SCFAs by psychrophilic processes, i.e., processes running at temperatures below 20 °C [31, 34]. Low temperatures significantly limit the growth rate of methanogens and thus promote the accumulation of SCFAs [35].

### Substrate composition as determinant of the SCFAs profile

Feedstock composition in terms of the component proteins, carbohydrates and lipids determines the ultimate SCFAs profile since the metabolic mechanisms to produce carboxylic acids differ from one component to another. Feedstock composition is selective for a substrate-adapted microbial community specialized in the hydrolysis and acidogenesis of specific compounds. Although bacteria belonging to the Firmicutes phylum have been identified as key microorganisms to maximize SCFAs- formation, the specific genera within the phylum highly depend on substrate composition, thereby determining the SCFAs profile [30, 36]. Table 1 presents different substrates, mainly derived from side and waste products that have been successfully used for SCFAs production via AF and the prevailing SCFAs obtained from the respective substrates.

**Table 1:** Possible feedstock for SCFA production, prevailing acids in the SCFA pool, and main operational parameters

Feedstock	Reactor type	Inoculum source	Operational conditions	Prevailing SCFA	References
Cheese whey	AnSBR^a^	From AD of cheese whey	30 °C, pH = 5, OLR = 6 g COD/Ld, HRT = 2 d	Acetic, propionic, butyric, valeric acids	[37]
Sewage sludge	CSTR^b^	Sewage sludge	37 °C, pH = 5.6, OLR = 0.9 g COD/Ld, HRT = 10 d	Acetic, propionic, iso-butyric, butyric, iso-valeric, valeric acids	[38]
Tuna waste	CSTR^b^	From AD of tuna waste	37 °C, pH = 5–10, OLR = 4 g COD/Ld, HRT = 10 d	Acetic, propionic, butyric, iso-valeric acids	[28]
Brewery wastewater	CSTR^b^	From AD of brewery wastewater	30 °C, pH = 5, OLR = 6 g COD/Ld, HRT = 2 d	Acetic, propionic, butyric, valeric acids	[39]
Cheese whey + brewery wastewater (different ratios)	UASB^c^	From AD of cheese whey	30 °C, pH = 5, OLR = 6 g COD/Ld, HRT = 2 d	Acetic, propionic, butyric, valeric acids	[40]
Cheese whey + sewage sludge (different ratios)	CSTR^b^	From AD of brewery wastewater	37 °C, pH = 5.5, OLR = 1–2.4 g COD/Ld, HRT = 15 d	Caproic, acetic, butyric, iso-butyric acids	[41]
Agroindustrial wastes: potato solid waste, grape marc distilled, grape marc, brewery spent grain	Batch		30 ºC	Acetic, butyric, propionic acids	[42]
Agroindustrial waste: Cucumber Tomato Lettuce	CSTR^b^	From a conventional AD of sewage sludge at 35 ºC	25 °C, pH = 3.8–4.6 (NO pH control), OLR = 3 g VS/Ld	Butyric, caproic acids Acetic, butyric acids Acetic, butyric acids	[27]
Agroindustrial waste: Cucumber Tomato Lettuce	CSTR^b^	From a conventional AD of sewage sludge at 35 ºC	25 °C, pH = 5.5–6 (pH control), OLR = 3 g VS/Ld, HRT = 9.3–10.2 d	Acetic, butyric, caproic acids Acetic, butyric, caproic acids Acetic, butyric acids	[25]
Agroindustrial waste: melon and watermelon	CSTR^b^	From a conventional AD of sewage sludge at 35 ºC	25 °C, pH = 5.5–6 (pH control), OLR = 3 g VS/Ld, HRT = 27 d and 20d	Iso-butyric, caproic acids	[30]
Microalgae biomass (Pretreated)	CSTR^b^	From a conventional AD of sewage sludge at 35 ºC	25 °C, pH = 5.7–6.3 (NO pH control), OLR = 1.5 g COD/Ld, HRT = 8 d	Acetic, propionic acids	[43]

^a^Anaerobic sequencing batch reactor

^b^Continuous stirred tank reactor

^c^Up-flow anaerobic sludge blanket

Such as temperature, *HRT* Hydraulic retention time, *OLR* Organic loading rate, *COD* chemical oxygen demand, *VS* Volatile solids, *d* Day if stated, *AD* Anaerobic digestion

#### Protein-rich substrates 

Such as sewage sludge or microalgae result in amino acid release. These amino acids are further converted into carboxylic acids via two main pathways: (i) Stickland fermentation in which, the amino acid serves as an electron acceptor giving rise to an SCFA with the same length as the original amino acid and (ii) amino acids reduction via Wood-Ljungdahl pathway, which requires the co-occurrence of hydrogen-utilizing bacteria. Previous studies focused on SCFAs production from proteinaceous substrates revealed acetic acid and propionic acid as the main SCFAs resulting from AF. For instance, Magdalena et al. (2019) [44] showed that 50% of SCFAs profile was dominated by these carboxylic acids when microalgae biomass was subjected to AF, while Llamas et al. (2021) [36] found that the same microalgae biomass resulted in a 60–65% of acetic and propionic acids dominance. Likewise, Iglesias-Iglesias et al. (2019) [42] showed that the presence of these SCFAs increased to 70–80% when sewage sludge was used as substrate. Although an SCFA mixture was produced in each study, acetic acid and propionic acid prevailed over other SCFAs, being further followed by butyric and valeric acids.

In fermentative processes, Firmicutes normally account for the highest relative abundance, covering from 65% to 83% of the microbial community. The high presence of amino acids as a result of protein degradation promotes within this phylum the growth of amino acids degraders such as *Peptostreptococcus*, *Sporoanaerobacter* or members of the order Clostridiales (especially *Clostridium*) [45]. *Peptostreptococcus* and *Sporoanaerobacter* produce propionic and acetic acid, respectively. On the other hand, *Clostridium* has been recognized as the only genus able to conduct Stickland fermentation under anaerobic conditions [46]. Thus, the presence of different amino acids degraders suggests that protein conversion into SCFAs is normally occurring by the co-existence of both metabolic mechanisms (Stickland and Wood-Ljungdahl pathway) when AF is performed by an open-mixed culture [43, 45].

SCFAs profiles attained via AF of carbohydrate-rich substrates follows a different trend since acetic acid and butyric acid become the most abundant carboxylic acids. Carbohydrate degradation results in a wide variety of sugars (fructose, glucose, galactose) that are metabolized into SCFAs. Although different metabolic pathways can be involved in this process, acetic- and butyric-type fermentations are the most common when AF is performed at mild operational conditions. Food waste has been one of the most studied carbohydrate-rich substrates to produce SCFAs given its large production worldwide. However, the carbohydrate content of FW varies depending on the location and seasonal features, which finally affects the SCFAs production efficiency and the acetic and butyric acid concentrations. For instance, Zhang et al. 2020 [47] found 40% of acetic acid dominance when potato peels were subjected to AF, whereas Greses et al. 2020 [25] identified butyric acid as a prevalent metabolic product (41.1 – 43.5%) during AF of cucumber and tomato residues. Recent studies about the effect of the substrate composition on SCFAs profile have determined that the higher the carbohydrate content, the higher the butyric acid concentration in the AF effluent [34].

Regarding the microbiome involved in carbohydrate degradation, bacteria belonging to the order Lactobacillales and Clostridiales (phylum Firmicutes) have been identified as key microorganisms in the process. Lactobacillales is a metabolically diverse order that involves lactic acid-producing bacteria as well as homoacetogenic bacteria. Whereas lactic acid can be further converted into SCFAs, homoacetogens are able to transform H_2_ and CO_2_ into acetic acid, increasing the abundance of this carboxylic acid [48].

In the case of AF of carbohydrate-rich substrates, *Clostridium tyrobutyricum*, *Acetobacterium woodii* and *Ruminococcus* are among the predominant bacteria. *C. tyrobutyricum* is known for its ability to transform lactic acid into butyric acid via butyrate-type fermentation [49], and *Ruminococcus* are involved in the degradation of carbohydrates into both butyric and acetic acid [27].

The bioconversion of organic matter into SCFAs is strictly dependent on the intrinsic nature of the organic material itself: typically, between 10% and 50% of COD in the feedstock can be converted into SCFAs, according to its accessibility and biodegradation rate [50].

It is important to highlight that although operational conditions can be tuned to maximize the hydrolytic activity of the microbiome to increase substrate degradation, some wastes are constituted by recalcitrant compounds that hamper microbial degradation, such as lignin. In this regard, lignocellulosic biomass (forest residues, herbaceous plants, wheat straw, rice straw, corn residues, and sugarcane bagasse) represents one of the most valuable resources to produce biochemicals given the large worldwide availability. However, the complex structure of these residues involves the use of pretreatments to increase the bioavailability of organic matter. The most common pretreatments (chemical catalysts, enzyme addition, steam explosion, physical disruption) require the addition of reagents and an energy input, which considerably affect the cost-effectiveness of the process [51].

## Extraction/purification of SCFAs

In order to make bioprocessed SCFAs available as platform chemicals, they must be obtained in adequate concentration and purification grade. Only in this case, their industrial use will be feasible [52, 53]. For this end, it is necessary to separate and concentrate the SCFAs from the fermentation broth. Recovery is, at the moment, the real bottleneck that limits the diffusion of bioprocesses for SCFAs production at the industrial scale. The advantages and disadvantages of existing technologies to separate and purify SCFAs have recently been reviewed and discussed [54]. The main techniques that have been considered for SCFAs recovery from fermentation broths in recent years are as follows:

(i) liquid − liquid extraction using organic solvents [55],
(ii) membrane separation [56, 57];
(iii) adsorption [53, 58–60];
(iv) distillation [61];
(v) electrodialysis [62, 63]

### Liquid − liquid extraction

Processes can be easily carried out and scaled up. These techniques are of primary interest in SCFAs recovery. Because of their chemical and physical characteristics, SCFAs can be transferred into liquid solvents such as kerosene and diesel (or biodiesel) under mild conditions like ambient temperature and pressure, relatively acidic pH and moderate shaking. On the other hand, the use of organic solvents involves some health and environmental risks. Organic solvents like kerosene and biodiesel can for example be used to recover SCFAs after distillation. A beneficial effect of trioctylphosphine oxide (TOPO) for optimizing SCFAs transfer under acidic conditions (pH 2.5) has been demonstrated by Alkaya et al. The higher the TOPO concentration in kerosene, the higher the SCFAs recovery [64].

### Membrane separation

Seems to have great potential for improvement due to the continuous discovery of new materials. Various ranges of filtration, such as nanofiltration, reverse osmosis, pervaporation, and membrane contactors, as well as membrane distillation for SCFAs separation and recovery of specific acids or acid groups, can be used. Recent studies showed high yield, high acid selectivity, and SCFAs recovery with low energy consumption and a small reactor footprint, making membrane-based separation processes more promising compared to other methods [59]. However, after fermentation, the liquid fraction of the fermentate is still rich in solids and colloids, which should be removed to avoid rapid fouling of the membranes. This requires more efficient pre-treatment (e.g., centrifugation, sieving) to reduce fouling problems and extend membrane module life [65].

### Ion exchange adsorption

In which the negatively charged carboxylic groups of SCFAs interact with positively charged groups such as amines, is highly selective. As with membrane separation, pretreatment to remove suspended solids and colloids from the liquid phase is of paramount importance to increase process efficiency and avoid poisoning of resin functional groups. In recent years, some researchers have investigated SCFAs adsorption efficiency for various solid matrices. Both powdered activated carbon (PAC) and various resin-like matrices (e.g., Lewatit VP OC 1065, Amberlyst A21) are effective in recovering SCFAs [53]. Da Silva and Miranda [66] studied the adsorption of single- and multi-component mixtures of SCFAs (acetic, propionic, and butyric) to purolite A133S (a tertiary amine-functionalized resin) and to granular activated carbon (GAC) and found that the resin gave 35% higher adsorption yields than GAC.

### Distillation

Is another possible option for the recovery of SCFAs [54]. During steam distillation, the fermentation broth is kept in an acidic condition (pH < 4) to protonate SCFAs and volatilize the molecules. The organic acids are then recovered with more than 98% efficiency in a basic solution. Moreover, this technique allows for the product fractionation because of different boiling point temperatures for SCFAs: different acids can be collected separately with a high purification grade [67].

### Electrodialysis

Allows for the recovery of SCFAs from fermentation broth. Previous studies showed the possibility of removing hundreds of mg/L of acetic, propionic, and butyric acids in the liquid phase [63]. In particular, acetic and propionic acids are effectively recovered and concentrated [62].

Overall, several techniques for recovering SCFAs are available; but they all need some optimization and improvement before they can be applied on a large scale. However, the portfolio of different techniques is extremely promising in this regard.

## Comparison of chemical synthesis and biosynthesis of SCFAs

A major difference between bioprocesses (e.g., AF) and catalytic chemical processes is that the latter usually require high temperatures (e.g., typically 200–250 °C) and pressures (e.g., up to 50–60 bar), while most bioprocesses take place at near room temperature and ambient pressure. Despite these considerations, and although most thermochemical processes are characterized by relatively high investment and operation costs, these chemical processes are still considered superior in terms of productivity, yields, and cost-effectiveness, though not in terms of sustainability. It has been reported that both thermochemical as well as biological conversions of feedstocks (e.g., biomass, waste) have drawbacks and that the former usually involves a high energy intake along with non-environmentally friendly solvent or catalyst addition, while the latter, bioprocess, has a lengthy cycle period and is less efficient in breaking down recalcitrant compounds [68]. Besides, thermochemical processes may be non-efficient for feedstocks with high moisture contents, although some recent developments have focussed on overcoming such issues, e.g., through supercritical water gasification. On the other side, bioprocesses may be affected by the presence of recalcitrant, hard to metabolize (e.g., lignin) compounds present in biomass or wastes, and pre-treatments may be required with the associated additional costs [69].

The applications of SCFAs obtained through AF are generally different from those derived from petroleum feedstocks. Biofuels or bioproducts of interest considered in anaerobic bioconversion processes include compounds such as biopolymers (PHA), microbial oils, and other biobased products. Petroleum-derived acetic acid is mainly used for the production of platform chemicals such as acetate esters, vinyl, ethyl, propyl, and butyl acetates, terephthalic acid, or acetic anhydride. It has applications in industrial sectors such as pharmaceuticals, polymers, food and beverage, paints, or coatings, among others. Bioproduction of acetic acid represents less than 10% of the world market and is mainly used for the production of vinegar [12, 13].

In short, the catalytic versus biological production of SCFAs is driven, among others, by fluctuating oil prices. When the latter is low, chemical processes are usually preferred, and when oil prices rise, bioprocesses regain interest. On the other hand, it is often difficult for bioprocesses to compete with the catalytic alternatives in terms of cost-effectiveness, although bio-based SCFAs are generally more environmentally friendly and they are produced under milder conditions, e.g., near room temperature and pressure, typical of anaerobic digestion processes for the production of biogas or SCFA [70]. In this sense, a recent work on LCA of SCFAs production from protein- and carbohydrate-rich organic wastes has clearly shown that biotechnologically produced acetic acid was more favourable than chemical synthesis [71]. Additionally, very low environmental impact corresponded to the scenarios producing SCFAs from agroindustrial wastes.

## SCFAs as carbon sources for bioproduction

### Microbial electrolytic cells (MECs)

Are an emerging technology for the production of H_2_. As the biological counter partner of water electrolysis, MECs have some advantages, such as the absence of oxygen (explosion risks) and the avoidance of KOH solution as used in alkaline water electrolysis cells. Because of that, MECs are considered more beneficial in terms of safety. At a technological level, a much lower cell voltage is needed in MECs, since the oxidation of organic matter can proceed at much lower voltages than those used in water electrolysis (1.23 V). MECs are based on a bioelectrochemical system that uses an external power source to convert an organic substrate into hydrogen by means of microbial catalysis. The oxidation of organic compounds is carried out by electroactive bacteria in the anode (bioanode), while H_2_ is produced by the reduction of protons in the cathode. Acetate is one of the most studied organic sources in MECs. The reactions that generate H_2_ using acetate are as follows:

$${\text{Anode}}\, CH_{{3}} COO^{ - } + {\text{ 4H}}_{{2}} O \to {\text{ 2HCO}}_{{3}}^{ - } + {\text{ 9H}}^{ + } + {\text{ 8e}}{-}$$

$${\text{Cathode}}\, {8}H^{ + } + { 8}e^{ - } \to { 4}H_{{2}}$$

$${\text{Total}}\, CH_{{3}} COO^{ - } + {\text{ 4H}}_{{2}} O \to {\text{ 2HCO}}_{{3}}^{ - } + H^{ + } + {\text{ 4H}}_{{2}}$$

Acetate provided the highest current densities [72] and H_2_ production rates [73] out of all substrates used. Similar to acetate, other acids, including butyric, propionic, valeric, and lactic acid, can also serve as electron donors with high efficiency after microbial adaptation to these new substrates [74]. In this way, it seems logical that anaerobic fermentation, which produces SCFAs, could be coupled with MEC with the ultimate aim of producing H_2_ from various wastes. In this type of bioelectrochemical system, acetic is preferred over butyric acid as an organic substrate for H_2_ production [75]. However, this statement might be misleading since not only the type of SCFAs is important for H_2_ production, but also the microbial systems might be crucial.

The microbial population and type of bacteria have a major impact on MEC performance. Common electrogenic communities employed in bioelectrochemical systems mainly include *Geobacter* and *Shewanella* [76]. In principle, these microbial systems form a biofilm in the anode that oxidizes the organic matter and transfers the electrons to the cathode. As a consequence of hydrogen production, the anode becomes acidic while the cathode becomes alkaline. The low pH surrounding the anode results in the inhibition of the microbial system as the bacteria are unable to cope with pH values far from neutral. The local acidification is a major problem in MECs, which requires the establishment of acidophilic electrogenic microbial systems. Considering this, AF has not only the potential to provide an alternative technology to generate innovative carbon sources (SCFAs) but also microorganisms that can thrive at slightly acidic pH. Indeed, AF is promoted at pH values in the range of 5.5 to 6.5 [27]. The use of microbial systems enriched with acidogens might be a promising solution for the further development of MECs. As previously mentioned, hydrolysis and acidogenesis in AF are mainly carried out by species belonging to the phylum *Firmicute*s, including species of the genera *Clostridium*, *Sporanaerobacter*, *Streptoccocus* and *Syntrophomonas*. These bacteria not only have the ability to ferment, but also exhibit an electrochemically active metabolism [77, 78]. It needs to be highlighted that AF is conducted by a microbial community and not by a pure bacterial strain. This fact is relevant to understand the limitations that MECs can exhibit. Studies that focus on electron transfer mechanisms usually use pure cultures. Axenic cultivations are easier to control but are also limited biologically by inherent traits. It is therefore conceivable that the use of AF-derived microbial communities, through their microbial diversity, could not only lead to more robust biosystems but also enhance their performance by exploiting complementary metabolic capabilities.

As a matter of fact, the coexistence of electroactive bacteria and hydrolytic and fermentative bacteria has been proven to be beneficial since other SCFAs (e.g., butyric acid) besides acetate may be oxidized, thereby improving MECs performance [79, 80].

Overall, the coupling of both bioprocesses (AF and MEC) can help unravel the suitable microbiomes for efficient MEC performance. Bacterial populations developing under AF conditions are adapted to slightly low pH values, and many of them are thought to be electroactive. In addition, the fermentate, which is rich in SCFAs, provides an excellent source of organic matter that can be used in MEC for hydrogen production. Moreover, the use of this type of effluent, rich in carboxylates such as SCFAs and other mineralized components, can have a positive impact on MEC functioning since the ionic conductivity is higher than what is normally encountered in wastewater (conventional organic source used in MECs).

### Microbial oils

Have been proposed as a good source of lipids for the production of oleochemicals that can replace petroleum or vegetable oils in various food and feed, chemicals, and fuel production processes. These microbial oils offer several advantages compared to using vegetable oils, such as the significantly shorter cultivation times of microorganisms and that cultivation does not require arable land. Microorganisms that are able to naturally accumulate more than 20% w/w of lipid per cell dry weight are referred to as oleaginous, and their lipid content can be increased under special cultivation conditions [81]. Sugars are the most commonly used carbon sources for lipid accumulation in oleaginous microorganisms and can add significant cost to the overall process. In this sense, considerable effort has been undertaken to find alternative low-cost substrates, which is necessary to establish economically viable oleochemicals production processes derived from microbial oils [82–85].

SCFAs produced in anaerobic fermentation processes from organic wastes can be metabolized by some oleaginous microorganisms, offering an interesting possibility to replace the sugar-platform in the oleochemical sector. Compared to sugars, SCFAs may trigger shorter metabolic pathways leading to higher conversion efficiencies [86]. However, metabolic pathways through which SCFAs are metabolized by oleaginous microorganisms are, in most cases, controversial. Thus, improving the knowledge of how microorganisms metabolize unusual substrates such as SCFAs is a fundamental issue to boost the transition to a more sustainable industry based on renewable raw materials.

Despite the identification of some oleaginous fungal strains that are a good choice for producing lipids from glycerol and lignocellulosic sugars [87–91], their ability to produce microbial oils from SCFAs is still poorly investigated.

Several *Bacillus* species, such as *Bacillus alcalophilus* and *Bacillus subtilis*, and some genera from the phylum Actinobacteria (*Mycobacterium*, *Streptomyces*, *Rhodococcus*, and *Nocardia*) can accumulate significant amounts of lipids [91]. Considering SCFAs as carbon source, *Rhodococcus* sp. is among the most promising bacteria for the production of microbial lipids. The *Rhodococcus* sp. YHY01 has recently been found to have lipid contents of up to 69% w/w when cultured on food waste-derived SCFAs [92]. Interestingly, when SCFAs such as propionic and valeric acids were metabolized by *Rhodococci*, odd-chain fatty acids (OCFAs) were enriched [93, 94]. These OCFAs are very interesting, valuable products with medical and nutritional applications [95, 96], and they are also alternatives to the precursors for the manufacture of chemicals. The ability of the oleaginous *Rhodococcus* strains to synthesize these OCFAs is an important trait that makes them very attractive compared to other oleaginous cell factories. For example, naturally occurring oleaginous yeasts produce relatively small amounts of OCFAs [97, 98]. Of all known oleaginous microorganisms, the oleaginous yeasts *Yarrowia lipolytica*, *Cutaneotrichosporon curvatum, Rhodotorula toruloides*, *Rhodotorula glutinis*, *Rhodotorula babjevae*, *Lipomyces starkeyi*, *Lipomyces lipofer* or *Williopsis saturnus* have demonstrated their ability to utilize SCFAs as a carbon source for microbial lipid production [89, 99, 100]. Other, less common yeasts species, like *Millerozyma farinosa*, *Trigonopsis cantarelli*, and *Geotrichum candidum* have been recently described as oil producers from SCFAs [101].

Nitrogen limitation and, consequently, a high carbon:nitrogen ratio promotes channeling of the carbon source towards lipid synthesis, thereby increasing lipid content in yeast cells. Acetic acid is one of the most commonly produced SCFA in AF, and its utilization for lipid production has been widely addressed in recent years [23, 100, 102]. Acetic acid is converted to acetyl CoA, which can directly be converted to fatty acids [103, 104]. Acyl-CoA can also be formed from organic acids other than acetic acid; for instance, propionyl-CoA is assumed to be the starting compound for the synthesis of odd-chain fatty acids in yeasts [104]. However, the metabolic pathways involved in the conversion of SCFAs (other than acetic acid) to microbial oils are still controversial, and recent research efforts are aimed at understanding the metabolism of SCFAs in yeast [105].

Despite the challenges associated with SCFAs utilization for microbial oil production, such as their toxicity, recent articles have demonstrated the suitability of organic waste-derived SCFAs as unusually low-cost carbon sources for the production of microbial lipids. In this sense, *Y. lipolytica* reached lipid contents of 26% w/w using SCFAs derived from food and vegetable waste [106], 23.3% w/w lipids in SCFAs-rich media derived from microalgae residual biomass [107], and up to 37.3% w/w lipids from SCFAs-rich digestates produced from agroindustrial wastes [23]. Furthermore, lipid yields obtained from SCFAs were comparable to or even higher than those obtained from sugar-based media, opening a new perspective for utilizing SCFAs derived from organic wastes as a cost-effective alternative to increasing the economic viability of the lipid production process. In this context, unraveling the capacity of yeast to efficiently convert SCFAs into products of interest would be crucial for an efficient implementation of the carboxylate platform [108].

### Polyhydroxyalkanoates (PHAs)

Are linear polymers of hydroxy acid residues connected by an ester bond. They are produced by a variety of microorganisms under conditions of excess carbon and limitation of essential nutrients, with the carbon source being accumulated as energy storage in the form of granular inclusions in the cell cytoplasm [109, 110]. Monomers of different lengths can be obtained depending on the microorganism and the carbon source used. This gives the biopolymers different properties [111]. PHAs have characteristics similar to conventional thermoplastics. The properties of PHAs can be very similar to those of petroleum-derived polymers, such as high melting temperature and high tensile strength [112]. The production of conventional plastics has exceeded 400Mt/year worldwide [113], and their end-of-life management is problematic. For this reason, PHAs, which are biodegradable products obtained from renewable sources, including carboxylic acids, have been investigated in recent years. When PHAs are degraded, the products generated are considered less or not environmentally harmful [114]. Because of their biodegradability, biocompatibility, and non-toxicity, PHAs have various uses, including agriculture, biological control agents, biofuels, medical applications, and others [115].

Currently, PHAs are produced on the pilot and industrial scale by pure cultures of PHA-accumulating bacteria using sugar- or fatty acid-containing carbon sources. These processes are quite costly due to the need to keep them sterile and the high substrate costs. Converting complex waste products using microbial consortia represents a novel research approach to the production of PHAs. The use of SCFAs derived from waste materials for PHA production is an innovative and environmentally friendly strategy that has received increasing attention in ongoing research in recent years [111, 116]. Table 2 shows some recent examples of converting waste-derived carboxylic acids as carbon sources for the production of PHAs. In terms of costs, PHA recovery involves an extraction and purification process which may lead to relatively high production costs, up to 2–3 USD per kg [117]. These costs are high in comparison with those of other biopolymers such as Poly-Lactic-Acid (PLA). PLA is derived from renewable resources such as corn starch, sugarcane, or cassava. However, the cost of disposal and the environmental impact of both polymers must be considered in a comprehensive cost analysis. While PLA production may have a somewhat lower cost compared to PHA production, the total cost should also take into account the environmental impact of disposal and the cost of waste management [118, 119].

**Table 2:** Typical feedstock and carbon sources for polyhydroxyalkanoates (PHAs) production

Feedstock	Description	Biomass	Carbon source (SCFAs)	Maximum PHA content of the biomass	References
Soft drink industrial wastewater	Uncoupled carbon and nitrogen feeding	MMC	acetic acid	25%	[120]
Fruit waste	Uncoupled carbon and nitrogen Feeding Strategy with different OLRs and SRTs	MMC	butyric acid > acetic acid > propionic acid	69%	[121]
Co-digestion of cheese whey and brewery wastewater	Influence of the feedstock mix ratio	MMC	acetic acid > butyric acid > propionic acid > valeric acid	50%	[40]
Co-digestion of cheese whey and sewage sludge	PHAs production from an effluent rich in caproic acid	MMC	caproic acid > acetic acid > butyric acid > valeric acid	32.5%	[41]
Wastewater of a potato-starch factory	A pilot-scale integrated into a food-industry effluent biological treatment	MMC	cetic acid > butyric acid	45%	[122]
Molasses waste	Study of pyruvate supplementation for the PHAs production	MMC	butyric acid > acetic acid > propionic acid	53.6%	[123]
Juices from fruit pulp	Production of a ter-polymer enriched in 3-hydroxyhexanoate (HHx)	MMC	caproic acid > butyric acid > acetic acid > valeric acid > propionic acid	71.3%	[124]
Cooked mussel processing wastewater	PHAs production at low pH and high salt	MMC	acetic acid > butyric acid > propionic acid > valeric acid, proteins	40.9%	[125]
Waste sludge	PHAs production with acid or alkaline sludge pretreatment	MMC	acetic acid > propionic acid > butyric acid > valeric acid	60.3%	[126]

The carbon sources (SCFAs) are listed in the order in which they were present relative to each other, as indicated by the "greater than" sign

*MMC* Mixed microbial cultures, *SCFAs* short-chain fatty acids

Similar to other applications based on SCFAs, the production of PHAs starts with anaerobic fermentation, during which PHA precursors such as SCFAs, lactate, and ethanol are produced from organic matter. The next step is culture selection, which aims to enrich and maximize the number of microorganisms of the mixed microbial cultures (MMC) capable of accumulating PHAs. The third step in PHA production is the accumulation phase, which focuses on maximizing the biopolymer yield by feeding the enriched culture with an excess carbon source (SCFAs and other compounds obtained from anaerobic fermentation) in the absence of nutrients [127].

During culture selection, cultivation conditions are applied that select organisms able to convert and accumulate the carbon source into PHAs as storage compounds. This is usually achieved by “aerobic dynamic feeding”, i.e., alternating “feast”- and “famine”- cultivation phases under aerobic conditions, where during the feast phase, a carbon surplus is provided, which can be stored by PHA-accumulating organisms. In the famine phase, carbon and energy limitation is applied, where PHA-accumulating organisms can utilize their intracellularly stored carbon to divide and outcompete other microbes. Lengths of feast and famine phases vary greatly between studies, dependent on the substrates and microbial consortia used. The feast phase must be long enough so that the carbon source is depleted, and the famine phase must allow a significant consumption of the accumulated PHAs [111, 116]. There are variations on this strategy, the most successful being “uncoupled carbon and nitrogen feeding”, in which nitrogen supply is limited during the feast phase, while there is an N-surplus during the famine phase [111, 116, 128]. Another selection strategy is anaerobic/aerobic enrichment. This strategy derives from biological phosphate removal by activated sludge in wastewater, where PHA accumulation in microbes was first observed. During anaerobic cultivation, polyphosphate and glycogen accumulating organisms accumulate PHAs, which are consumed during the aerobic phase. However, this strategy usually leads to lower PHA levels than aerobic dynamic feeding with uncoupled carbon and nitrogen feeding [116, 120]. Aerobic-anoxic dynamic feeding can also be used for culture selection. During the aerobic feast phase, PHAs are accumulated, and ammonium is oxidized to nitrite. The nitrite is then utilized as an electron acceptor in the anaerobic famine phase, using the PHAs as carbon sources. This strategy can be used for parallel enrichment of PHA-forming organisms and nitrogen removal from wastewater [116, 129, 130]. This aerobic dynamic feeding is the most common method for the accumulation of PHAs because of its higher yield compared to the other two methods.

The final step, PHA accumulation, is performed using a microbial consortium enriched for PHA accumulation (see above) under carbon excess with nitrogen or phosphate limitation. The feeding regime seems to be the most critical aspect during PHA accumulation since too high substrate concentrations should be avoided [116]. A variety of different batch-feeding regimes have been tested for different substrates. Pulsed fed-batch cultivation has been suggested for synthetic SCFA-substrates. However, fermentation effluents rarely have a high carbon source content. Continued on-demand feeding processes have been developed, using pH (as consumption of SCFAs increases pH) or dissolved oxygen as an indicator. Other cultivation parameters, including temperature, pH, and dissolved oxygen concentration, also play a role in PHA accumulation [116, 130]. Using different cultivation techniques and substrates, PHA concentrations of more than 70% per biomass could be reached from the waste substrate using mixed cultures (Table 2).

## Conclusions

Climate change, rain forest cutting, and current political developments show that there is an urgent necessity to replace fossil resources, and even renewable resources, such as vegetable oil, with sustainable alternatives. Due to their high versatility, the interest in SCFAs production has increased significantly in recent years. SCFAs have the potential to be used as platform chemicals or as a substrates for biotechnologically relevant microorganisms to generate biofuels, biochemicals and biomaterials.

These short-chain organic acids can be synthesized either chemically or biologically with the latter being the more economically friendly and greener alternative. The recovery of SCFAs is a fundamental step towards creating a new chemical platform where biomass instead of oil is the feedstock, and substantial progress has been made toward the bioproduction and valorization of SCFAs. Several economically sustainable techniques are available. A variety of demonstration plants has been established during the last years, and thus the establishment of industrial processes seems to be possible in the near future [131–136].

SCFAs can be bio-synthesized from mixed waste, such as sewage sludge or municipal residues. Thus, they have the potential to be the basis for developing local solutions, adding value to in part environmentally problematic waste products, which makes them advantageous over chemically synthesized SCFAs generated from fossil resources. The final concentration and proportions of biosynthesized SCFAs can be controlled by substrate composition and process conditions.

Clearly, research is still needed to optimize recovery, concentration and purification of SCFAs from fermentation broth. This is an essential step for the development of full-scale application of this approach.

Waste-derived SCFAs can be used efficiently by electrogenic bacteria in bioelectrochemical systems. Various types of microorganisms can produce microbial oils or other biodegradable products such as PHAs from SCFAs. Microbial oils can be sustainable precursors to various oleochemicals that can replace many petroderivatives. PHAs represent a promising biodegradable alternative to conventional plastics, which are otherwise derived from fossil fuels. The large-scale production of PHAs has made a breakthrough due to the use of mixed microbial cultures and waste-produced carboxylic acids as carbon sources. Production costs have decreased and their use has increased, due to the different characteristics of their specific composition.

However, generally the use of SCFAs produced from waste material on an industrial scale is still hampered by the cost of the associated processes. Nevertheless, the significant advances that have been made in recent years, e.g., in the optimization of fermentation processes and purification of SCFAs, together with an increasing awareness of the need to reduce dependence on fossil raw materials and edible plant materials, will inevitably lead to the establishment of corresponding biotechnological processes in the near future.

## Data Availability

Not applicable.

## Abbreviations

AF: Anaerobic fermentation
GAC: Granular activated carbon
GHG: Greenhouse gases
HRT: Hydraulic retention time
MEC: Microbial electrolytic cells
MMC: Mixed microbial cultures
OCFAs: Odd-chain fatty acids
OLR: Organic loading rate
PAC: Powdered activated carbon
PAOs: Polyphosphorus accumulating organisms
PHAs: Polyhydroxyalkanoates
SCFAs: Short-chain fatty acids
TOPO: Trioctylphosphine oxide
